# Combining three independent pathological stressors induces a heart failure with preserved ejection fraction phenotype

**DOI:** 10.1152/ajpheart.00594.2022

**Published:** 2023-02-10

**Authors:** Yijia Li, Hajime Kubo, Daohai Yu, Yijun Yang, Jaslyn P. Johnson, Deborah M. Eaton, Remus M. Berretta, Michael Foster, Timothy A. McKinsey, Jun Yu, John W. Elrod, Xiongwen Chen, Steven R. Houser

**Affiliations:** ^1^Cardiovascular Research Center, Lewis Katz School of Medicine, Temple University, Philadelphia, Pennsylvania, United States; ^2^Department of Biomedical Education and Data Science, Center for Biostatistics and Epidemiology, Lewis Katz School of Medicine, Temple University, Philadelphia, Pennsylvania, United States; ^3^Cardiovascular Institute, Perelman School of Medicine, University of Pennsylvania, Philadelphia, Pennsylvania, United States; ^4^Division of Cardiology, Department of Medicine, University of Colorado Anschutz Medical Campus, Aurora, Colorado, United States; ^5^Consortium for Fibrosis Research and Translation, University of Colorado Anschutz Medical Campus, Aurora, Colorado, United States; ^6^Department of Cardiovascular Sciences, Center for Metabolic Disease Research, Lewis Katz School of Medicine, Temple University, Cardiovascular Research Center, Philadelphia, Pennsylvania, United States; ^7^Department of Cardiovascular Sciences, Center for Translational Medicine, Lewis Katz School of Medicine, Temple University, Cardiovascular Research Center, Philadelphia, Pennsylvania, United States; ^8^Tianjin Key Laboratory on Technologies Enabling Development of Clinical Therapeutics and Diagnostics, School of Pharmacy, Tianjin Medical University, Tianjin, China

**Keywords:** cardiac hypertrophy, heart failure with preserved ejection fraction, histone deacetylases, M_2_-macrophage, suberoylanilide hydroxamic acid

## Abstract

Heart failure (HF) with preserved ejection fraction (HFpEF) is defined as HF with an ejection fraction (EF) ≥ 50% and elevated cardiac diastolic filling pressures. The underlying causes of HFpEF are multifactorial and not well-defined. A transgenic mouse with low levels of cardiomyocyte (CM)-specific inducible Cavβ2a expression (β2a-Tg mice) showed increased cytosolic CM Ca^2+^, and modest levels of CM hypertrophy, and fibrosis. This study aimed to determine if β2a-Tg mice develop an HFpEF phenotype when challenged with two additional stressors, high-fat diet (HFD) and *N*^ω^-nitro-l-arginine methyl ester (l-NAME, LN). Four-month-old wild-type (WT) and β2a-Tg mice were given either normal chow (WT-N, β2a-N) or HFD and/or l-NAME (WT-HFD, WT-LN, WT-HFD-LN, β2a-HFD, β2a-LN, and β2a-HFD-LN). Some animals were treated with the histone deacetylase (HDAC) (hypertrophy regulators) inhibitor suberoylanilide hydroxamic acid (SAHA) (β2a-HFD-LN-SAHA). Echocardiography was performed monthly. After 4 mo of treatment, terminal studies were performed including invasive hemodynamics and organs weight measurements. Cardiac tissue was collected. Four months of HFD plus l-NAME treatment did not induce a profound HFpEF phenotype in FVB WT mice. β2a-HFD-LN (3-Hit) mice developed features of HFpEF, including increased atrial natriuretic peptide (ANP) levels, preserved EF, diastolic dysfunction, robust CM hypertrophy, increased M_2_-macrophage population, and myocardial fibrosis. SAHA reduced the HFpEF phenotype in the 3-Hit mouse model, by attenuating these effects. The 3-Hit mouse model induced a reliable HFpEF phenotype with CM hypertrophy, cardiac fibrosis, and increased M_2_-macrophage population. This model could be used for identifying and preclinical testing of novel therapeutic strategies.

**NEW & NOTEWORTHY** Our study shows that three independent pathological stressors (increased Ca^2+^ influx, high-fat diet, and l-NAME) together produce a profound HFpEF phenotype. The primary mechanisms include HDAC-dependent-CM hypertrophy, necrosis, increased M_2_-macrophage population, fibroblast activation, and myocardial fibrosis. A role for HDAC activation in the HFpEF phenotype was shown in studies with SAHA treatment, which prevented the severe HFpEF phenotype. This “3-Hit” mouse model could be helpful in identifying novel therapeutic strategies to treat HFpEF.

## Graphical abstract

**Figure:**
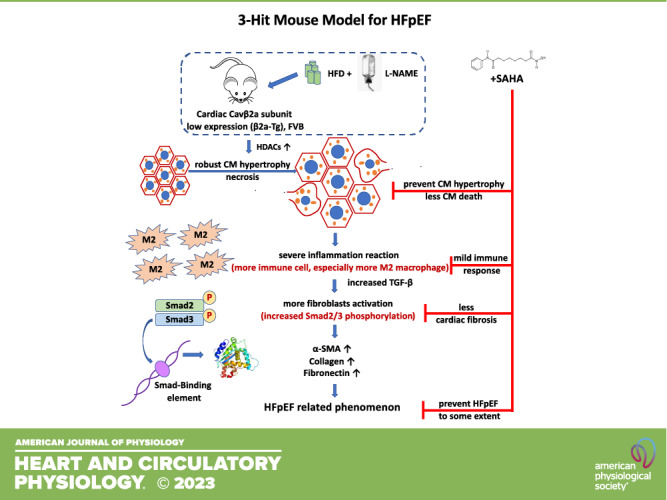

## INTRODUCTION

Heart failure (HF) with preserved ejection fraction (HFpEF) is a complex clinical syndrome defined as HF with left ventricular ejection fraction ≥50% with elevated LV filling pressures at rest or during exercise (1). It is a major public health problem (2). HFpEF has a high prevalence of 1.1%–1.5% in the general population and accounts for ∼50% of all HF cases. HFpEF prevalence is rising by ∼1% per year, likely because of an aging population and ongoing epidemics of hypertension, obesity, and diabetes mellitus (3–6). HFpEF is also characterized by high morbidity and mortality. After hospitalization for HF, the 5-yr survival rate of HFpEF is 50% (7), and every second patient reenters the hospital within 6 mo after the previous hospitalization (8). Better understanding and treatment of this disorder are clearly needed.

HFpEF is recognized as a multiorgan, systemic syndrome (4) in which cardiac, pulmonary, renal, skeletal, immune, inflammatory, metabolic, and other components combine to cause symptoms and disease (9). The cellular and molecular mechanisms that underlie this syndrome are not well understood, but the HFpEF cardiac phenotype includes cardiac hypertrophy, myocardial fibrosis, Ca^2+^ signaling pathway defects, inflammation, mitochondrial, and metabolic defects (4, 9, 10). Animal models that recapitulate critical HFpEF features are needed to better understand HFpEF and identify targets for novel therapies.

HFpEF animal models include Dahl salt-sensitive rats (11), spontaneously hypertensive rats (12), mice and rats with aortic constriction (13), aging models (14), aortic-banded cats (15), DOCA and salt-loaded pigs receiving a high-fat diet (16), and *N*^ω^-nitro-l-arginine methyl ester (l-NAME) plus high-fat diet (HFD) mice (17). Although some multifactorial models develop features of human HFpEF, most of the preclinical animal HFpEF models fail to meet the HFpEF clinical criteria like heart failure association pretest assessment, echocardiography and natriuretic peptide, functional testing, Final etiology (HFA-PEFF) diagnostic algorithm (18). The HFA-PEFF (heart failure association pretest assessment, echocardiography and natriuretic peptide, functional testing, Final etiology) algorithm is a stepwise approach based on expert consensus to establish diagnosis in patients with suspected HFpEF (19). The HFA-PEFF score is determined by scoring the natriuretic peptide levels and the echocardiographic findings of cardiac function and structure. If the HFA-PEFF score is ≥5 points, HFpEF is diagnosed, and if the HFA-PEFF score is ≤1 point, HFpEF could be excluded. If the HFA-PEFF score is between 2 and 4 points, the diagnosis for HFpEF needs further evaluation. Preclinical animal models with critical HFpEF features could help to define the complex HFpEF pathophysiology and to test putative HFpEF treatments. To date, there is only one successful clinical trial in HFpEF (20), and additional treatments are needed (1).

The aim of the current study was to characterize a novel mouse model that combines three pathological stressors: *1*) increased calcium (Ca^2+^) influx caused by cardiomyocyte (CM)-specific expression of the l-type Ca^2+^ channel (Cav) β2a-subunit (β2a-Tg mice), *2*) high-fat diet (HFD), and *3*) l-NAME (LN, nitric oxide synthase inhibitors) (termed the 3-Hit model). Schiattarella et al. (17) were the first to present a 2-Hit (HFD and l-NAME) mouse model with the C57BL/6 strain that developed features of human HFpEF. They observed that mice subjected to both stress factors developed a HFpEF phenotype, including lung congestion and reduced exercise tolerance with increased natriuretic peptides. However, this profound HFpEF phenotype was not observed in FVB wild-type (WT) mice subjected to HFD and l-NAME for 4 mo.

Our results show that in FVB WT mice, all three stressors needed to be present to induce phenotypic features of human HFpEF. This study also shows that the expression of histone deacetylases (HDACs, CM hypertrophy regulators) is increased in 3-Hit mice and is associated with CM hypertrophy and cell necrosis, followed by an increased cardiac M_2_-macrophage population, fibroblast activation, and myocardial fibrosis. The HDAC inhibitor suberoylanilide hydroxamic acid (SAHA) reduced the HFpEF phenotype in 3-Hit mice.

## METHODS

All experiments involving animals conformed to the Guide for the Care and Use of Laboratory Animals published by the National Institutes of Health (NIH Publication, 8th ed., Revised 2011) and were approved by the Temple University Institutional Animal Care and Use Committee. In addition, the studies complied with all ethical regulations.

### Experimental Animals

FVB mouse strain was used in our study. Cardiac myocyte specific [α-myosin heavy chain (MHC) promoter] with inducible tetracycline-activator (tTA) expression of the β2a-subunit of L-type Ca^2+^ channel (Cavβ2a) was used. This β2a-Tg with relatively low expression level was documented (Supplemental Fig. S3; all Supplemental material is available at https://www.doi.org/10.6084/m9.figshare.22068815) (21). The overexpression of β2a-subunit increases the open probability and membrane trafficking of the pore-forming Cav1.2α1c-subunit, which further increased Ca^2+^ influx in cardiomyocytes as previously reported (21, 22).

The β2a-Tg mice were established with the inducible (tet-off), bitransgenic system (22) (Supplemental Fig. S3). Mice with the tetracycline transactivator (tTA) driver gene and the Cavβ2a gene (double transgenic, DTG) without the doxycycline-containing chow were used as our β2a-Tg experimental group. Doxycycline is a derivative of tetracycline and hence represses β2a transgene expression. The Cavβ2a transgene was not expressed until adulthood to avoid developmental complications (22). For each litter, β2a-Tg mice were separated into different treatment cohorts, and their WT mice littermates were separated into corresponding treatments as well. Sex-matched animals (β2a-Tg and WT mice) were given different treatments at the age of 4 mo when the Cavβ2a gene had been fully expressed.

### Special Diet and Water Treatment

Both WT and β2a-Tg mice were housed in an animal room with a 12-h:12-h light/dark cycle from 6:00 am to 6:00 pm, a temperature of 22 ± 3°C, relative humidity of 50 ± 6%, and free access to food (Cat. No. 2916, Teklad for normal chow diet groups and D12492, Research Diet for the high-fat diet groups) and water [tap water, or *N*^ω^-nitro-l-arginine methyl ester; 0.5 g/L, Cayman Chemical], or l-NAME with suberoylanilide hydroxamic acid (SAHA, vorinostat, 670 mg/L, Biogems; 670 mg SAHA dissolved by 6.7 mL DMSO and 20 mL PGE300) (Detailed study groups provided in Supplemental Table S1).

### Study Design

Four-month-old sex-matched WT and β2a-Tg mice were given either normal chow (WT-N and β2a-N) with normal water or a high-fat diet (HFD) with/without l-NAME in water (WT-HFD, WT-LN, WT-HFD-LN, β2a-HFD, β2a-LN, and β2a-HFD-LN) for 4 mo. SAHA treatment was given to another group of 4-mo-old sex-matched β2a-Tg mice together with HFD and l-NAME (β2a-HFD-LN-SAHA) for 4 mo as well. Echocardiography was performed at baseline (4 mo old, before treatment) and once a month after different treatments. Long-term animal survival and body weight (BW) were recorded during the 4-mo follow-up (Supplemental Fig. S1).

After 4 mo of treatment, mice were terminated using inhaled isoflurane (Butler Shein Animal Health, Dublin Ohio) after measurement of hemodynamics parameters. BW weight was recorded at terminal time points. Organs, including heart, lung, liver, kidney, and spleen, were carefully trimmed and collected, rinsed with PBS, and weighed after blotting off the excess fluid. Organ weight-to-body weight ratios were calculated. Heart tissues were excised and cut into two parts (basal and apical). The basal part containing part of papillary muscles was fixed with 10% formalin, then paraffin embedded for histology following previously described protocols (23, 24). The apical part was snap-frozen in liquid nitrogen for molecular analysis. Plasma samples were collected for further study. The analysis of histology such as cardiomyocyte cross-sectional area (CSA), Picrosirius red staining, and echocardiography was performed by investigators blinded to groups.

### Echocardiography in Animal Study

Transthoracic echocardiography was performed using a Vevo2100 ultrasound system (VisualSonics; Toronto, ON, Canada). In brief, mice were placed in the supine position on a heated platform with all legs taped to electrocardiographic electrodes for recording. Mice were initially anesthetized with 2% isoflurane and then 1% during the ultrasound procedure to maintain a heart rate between 450 and 500 beats/min. The mouse’s body temperature was maintained within a range of 37.0 ± 0.5°C. Hair was removed from the chest using chemical hair remover before imaging.

Images were obtained in the short-axis B-mode, long-axis B-mode, and M-mode at the level of the midpapillary muscles for analysis of systolic function and dimensions. Parameters include diastolic left ventricular anterior wall thicknesses (LVAWd), end-diastolic left ventricular posterior wall thickness (LVPWd), end-diastolic left ventricular internal diameter (LVIDd), LV ejection fraction (LVEF), LV fractional shortening (LVFS), and left atrial diameter (LA). Diastolic function was determined using B-mode at parasternal long-axis view and apical four-chamber view, pulsed-Doppler (PW), and tissue-Doppler imaging (TDI) as previously described (23, 25). The left atrial diameter was measured at the parasternal long-axis view. PW was used to obtain the mitral inflow *E*, and TDI was used to measure the *e*′ wave. *E*/*e*′ was then calculated. Long-axis and short-axis B-mode images were collected for speckle-tracking strain analysis. Parameters were measured offline with VevoLab v3.2.6 (VisualSonics).

### Invasive Hemodynamics (In Vivo Intra-LV Pressure Measurements)

Invasive hemodynamics were performed after 4 mo of treatment. Briefly, intra-LV pressure was measured with a 1.4-Fr Millar pressure catheter (SPR-1000, Millar Instruments, Houston, TX) connected to an AD Instruments Power-Lab 16/30 (ADInstruments, Colorado Springs, CO) with LabChart Pro 7.0 software as previously reported. After mice were anesthetized with 2% isoflurane to maintain HR in the 450–500 beats/min range, a neck incision along the midline was made, and the right carotid artery was exposed. The pressure catheter was inserted into the right common carotid artery and advanced into the left ventricular (LV) chamber to measure left ventricular pressures and volumes. Blood pressure was recorded when the catheter was in the right common carotid artery. After entering in the LV, the catheter was carefully adjusted to avoid direct contact with the ventricular wall so that smooth intra-LV pressure traces could be clearly recorded. Data were analyzed offline with the blood pressure module in the LabChart7.0 software.

### Histology and Immunofluorescence Staining

Four to six mice were included in each group. After the mice were euthanized, the hearts were excised and cut into two parts (basal and apical). The basal portion containing part of the papillary muscles was fixed in 10% neutral buffered formalin and then embedded in paraffin. Three 5-μm-thick sections of basal pieces (one section per piece) were sliced, deparaffinized (xylene, Fisher Scientific, Fair Lawn, NJ), dehydrated (100%, 95%, 70%, and 50% ethanol, sequentially; Fisher Scientific, Fair Lawn, NJ) for the following staining:

1. Wheat-germ agglutinin (WGA, Life Technologies W11261, 1:100) (26) and nuclei (4′,6-diamidino-2-phenylinodole, DAPI, 268298 Millipore, 1:1,000) (26) staining was used to determine cardiomyocyte cross-sectional area (CSA). Images were taken using Nikon Eclipse Ti Confocal microscope, and at least 12 fields of view were taken of the left ventricle from three sections of the heart. Cardiomyocyte CSA was analyzed using NIH ImageJ software.
2. Interstitial fibrosis was detected by Picrosirius red staining using a kit (ab150681, Abcam). Von Kossa staining was performed to detect Ca^2+^ deposits according to the manufacturer’s protocol (ab150687; Abcam). Pictures were taken using a Nikon Eclipse Ti Confocal microscope with DS-Ri2 light camera. At least 15 views from each animal that did not include vessels were analyzed. Fibrosis (red) and nonfibrosis (pink) areas were calculated with the “color threshold” tool from ImageJ software (v.1.49v; NIH).
3. Terminal deoxynucleotidyl transferase dUTP nick end labeling (TUNEL) staining was performed with the DeadEnd Fluorometric TUNEL System kit (Promega, Madison, WI) as previously described (27). Briefly, after being deparaffinized and dehydrated, the slides were then fixed in 4% paraformaldehyde solution in PBS (Affymetrix, Cleveland, OH). After being washed in PBS, the tissue was digested by incubation with the Proteinase K included in the Promega kit for 8 min as per protocol instructions. Slides were then washed and incubated with the labeling cocktail for 1 h at 37°C. The reaction was then stopped with the included SSC solution, and the slides were washed. The slides were fixed once more with paraformaldehyde before α-sarcomeric actin (Sigma, A2172, 1:500, RRID: AB_476695) (26), WGA, and DAPI immunofluorescence staining. Images were taken using Nikon Eclipse Ti Confocal microscope, and at least 12 fields of view were taken of the left ventricle using a ×20 objective. NIS Confocal analysis software was used to analyze the images.
4. When determining the immune response among different groups, primary antibodies anti-CD45 (AF114, R&D Systems, 1:100, RRID: AB_442146) (28), anti-CD68 (MAB10114, R&D Systems, 1:100, RRID: AB_621929), anti-CD206 (AF2535, R&D Systems, 1:100, RRID: AB_2063012), anti-α-smooth muscle actin (ab5694, Abcam, 1:100, RRID: AB_2223021), anti-protein disulfide isomerase/P4HB (NB 300-517, Novus Biologicals, 1:100, RRID: AB_531260) (29), and anti-phospho-Smad2-S465/467 + Smad3-S423/425 (AP0548, ABclonal, 1:100, RRID: AB_2771541) (30) antibody was used for immunofluorescent staining of heart tissues. The secondary antibodies (26) were rhodamine Red-X (RRX) AffiniPure donkey anti-goat IgG (705-295-147, 1:100, RRID: AB_2340423), rhodamine (TRITC) AffiniPure donkey anti-rabbit IgG (711-095-152, 1:100, RRID: AB_2315776), rhodamine Red-X (RRX) AffiniPure donkey anti-mouse IgM (715-295-020, 1:100, RRID:AB_2340829), and fluorescein (FITC) AffiniPure donkey anti-rabbit (711-095-152, 1:100, RRID:AB_2315776) from Jackson ImmunoResearch. Nuclei were stained with DAPI.

### Quantitative Real-Time PCR

Total RNA was extracted from snap-frozen myocardial tissue using miRNeasy Mini kit (Qiagen) following the manufacturer’s instructions and then digested with DNase I (18068, Invitrogen). cDNA was synthesized with SuperScript III first strand (18068, Invitrogen) as previously described (25, 26). Real-time PCR was performed using the Quantifast Sybrgreen PCR kit (204057, Qiagen) and the QuantStudio 3 Real-Time PCR System (A28567, Thermo Fisher). *Ct* values were normalized with respect to β_2_-microglobulin (β_2_M). Fold changes were calculated with respect to WT-N mice compared with different treatment groups. Fold changes were calculated with respect to HDAC1 when compared among different HDACs.

The following primer sets were used (forward, reverse):

β_2_M, 5′- ATGTGAGGCGGGTGGAACTG, 5′- CTCGGTGACCCTG GTCTTTCTG; atrial natriuretic peptide (ANP), 5′- GCCCTGAGTGAGCAGACTG, 5′- GGAAGCTGTTGCAGCCTA; HDAC1, 5′- GTCCGGTGTTTGATGGCTTG, 5′- GCAGTGGGTAGTTCACAGCA; HDAC2, 5′- TATCCCGCTCTGTGCCCTAC, 5′- GAGGCTTCATGGGATGACCC; HDAC3, 5′- GACTTCTACCAGCCGACGTG, 5′- GCTTCTGGCCTGCTGTAGTT; HDAC8, 5′- CTGGACATACTTGACCGGGG, 5′- ACCGCTTGCATCAACACACT; HDAC4, 5′- GGGAGCAGCATCATGGTTCAA, 5′- TGAGAACTGGTGGTCCAAGC; HDAC5, 5′- AGAGTGACGTCTCCGAATGTTG, 5′- AGGAGTCCGTGGCAGGATTT; HDAC6, 5′- AGATCTGCGCGAGTGGAAG, 5′- CTCTCTGATGGCATGGAGCC; HDAC7, 5′- TATTCCCTACAGCCTGCCCACT, 5′- ACAGTGGGGCATGAGAGACT; HDAC9, 5′- CCATTGCCACGTGAACAACC, 5′- GACGACAGGATCCACCACAG; TGF-β, 5′- GCCCGAAGCGGACTACTATG,5′-TTTGGGGCTGATCCCGTTG; FN1, 5′- AGAAGACAGGACAGGAAGCTC, 5′- ATGGCGTAATGGGAAACCGT; LOX, 5′- TTCCAAGCTGGTTTCTCGCC, 5′- GTCCGATGTCCCTTGGTTCT; MMP9, 5′- CGCTCATGTACCCGCTGTAT, 5′- TGTCTGCCGGACTCAAAGAC.

### Western Blot Analysis

Lysates from snap-frozen heart tissues were prepared and analyzed using Western blot analysis as previously described (23). The following primary antibodies were used: GAPDH (EMD Millipore Cat. No. MAB374, 1:1,000, RRID: AB_2107445) (21), anti-CACNB2 (calcium voltage-gated channel auxiliary subunit-β2) (A16037, ABclonal, 1:1,000, RRID:AB_2763475) (31). The following secondary antibodies were used: 800CW donkey anti-rabbit (Cat. No. 926-32213, 1:5,000, RRID: AB_2715510) (26) and 680RD donkey anti-mouse (Cat. No. 926-68072, 1:5,000, RRID: AB_2814912) (26) purchased from LICOR (Lincoln, NE). Briefly, protein lysates were prepared from heart tissues, followed by denaturation with 12% SDS and derivatization with 1× DNPH (2,4-dinitrophenylhydrazine). Derivatized protein samples (10 μg/well) were used for Western blot analysis and immunodetection. The gel used is Mini-PROTEAN TGX Precast Gels (Cat. No. 456-1086, Bio-RAD, 4–15%). Western blot band intensities were quantified using Li-Cor Image Studio computer software.

### Statistical Analysis

Data are represented as means ± SE. The distributions of all continuous variables were tested for normality assumptions using the normal probability plot along with the Anderson–Darling normality test using GraphPad Prism. For parameters with a single measurement between two groups in the animal study, group comparisons were performed using the two-sample *t* test or the Mann–Whitney *U* test, depending on the data distribution. For parameters with a single measurement among multiple groups, the difference was evaluated using one-way ANOVA followed by the Tukey post hoc multiple comparison test. For body weight data and echocardiography parameters with repeated measures over time, linear mixed-effects models were used to estimate mean values at each assessment time point and to test treatment group differences at each time point as well as change versus baseline over time within each treatment group. In each linear mixed-effects model, time and treatment group were included as fixed effects along with its time-by-treatment group interaction term. Pairwise comparisons between various experimental groups under these mixed-effects models were performed using the Tukey post hoc multiple comparison tests. For in vivo data among groups under multiple treatments, the analysis was performed by two-way ANOVA, followed by the Tukey multiple comparisons test. Two-sided testing was used for all statistical comparisons. A *P* value of <0.05 was considered statistically significant. Data analyses were performed using the GraphPad Prism software (v.8.4.3, GraphPad Inc, La Jolla, CA) and/or SAS (v.9.4, SAS Institute Inc., Cary, NC).

## RESULTS

### Effects of High Fat Diet, l-NAME, and HFD + LN in WT Mice

**Figure 1.**
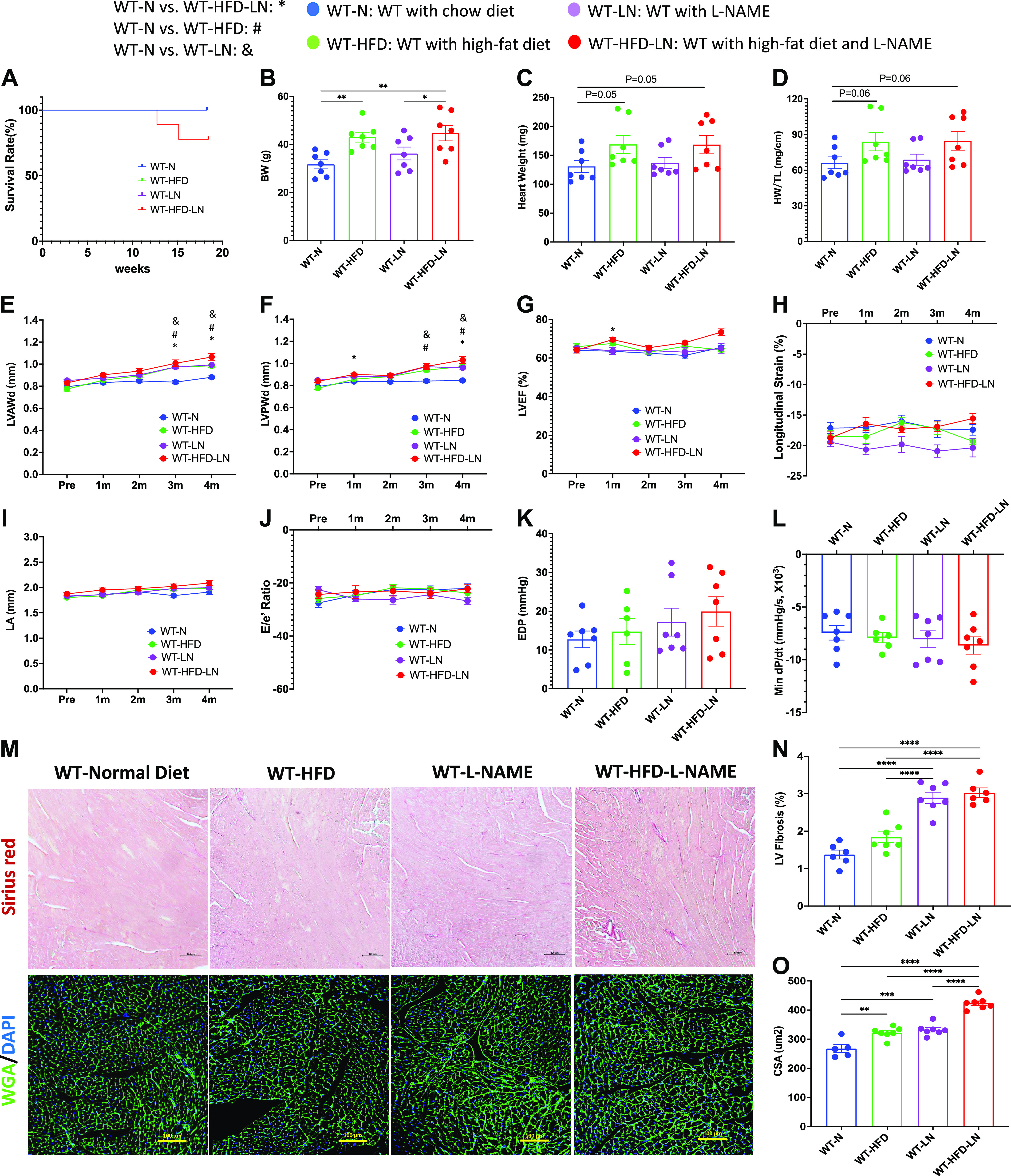
Effects of high-fat diet (HFD), *N*^ω^-nitro-l-arginine methyl ester (l-NAME, LN), or HFD + l-NAME on the heart failure with preserved ejection fraction (HFpEF) phenotype in wild-type (WT) mice. *A*: survival rate from 4-mo follow-up. Body weight (BW; *B*), heart weight (HW; *C*), and ratio of HW to tibia length (HW/TL; *D*) at the time of euthanasia. Conventional and sophisticated echocardiography data showing left ventricular (LV) wall thickness (*E* and *F*), LV ejection fraction (LVEF; *G*), LV longitudinal strain (*H*), left atrium (LA) diameter (*I*), and ratio between early mitral inflow velocity (*E*) and mitral annular early diastolic velocity (*e′*) (*E*/*e*′; *J*). Hemodynamics data showing LV end-diastolic pressure (LVEDP; *K*) and maximum rate of pressure decay (dP/d*t*_min_; *L*). *M*: representative images of hearts stained with Picrosirius red and wheat germ agglutinin (WGA). *N*: quantification of the percentage of Picrosirius red-positive area. *O*: quantification of cardiomyocyte cross-sectional area (CSA). DAPI, 4′,6-diamidino-2-phenylindole; LVAWd, end-diastolic left ventricular anterior wall thicknesses; LVEF, left ventricular ejection fraction; LVPWd, end-diastolic left ventricular posterior wall thickness; N, normal chow diet. Data shown are means ± SE. Tukey post hoc multiple comparison adjusted *P* values: *E–J*: *P* < 0.05, *WT-N vs. WT-HFD-LN, #WT-N vs. WT-HFD, and &WT-N vs. WT-LN; and other panels: *P < 0.05, **P < 0.01, ***P < 0.001, ****P < 0.0001. Total number of animals (*n*) and number of females and males included in each group are reported in the Supplemental Table.

Four-month-old FVB wild-type (WT) mice were fed with either a normal chow diet (WT-N) or treated with a HFD and/or l-NAME in water (WT-HFD, WT-LN, and WT-HFD-LN) for 4 mo (Supplemental Fig. S1). HFD treatment caused an increase in both body weight (BW) and blood pressure, while l-NAME treatment increased the blood pressure of WT mice (Fig. 1*B*; Supplemental Fig. S2, *A* and *B*). There was no significant difference in survival rate among the four groups (Fig. 1*A*). Four months of HFD and/or l-NAME treatment led to a trend to increase heart weight (HW), but not significantly higher HW to body weight ratio (HW/BW) and HW to tibia length ratio HW/TL (Fig. 1, *C* and *D*; Supplemental Fig. S2*C*) compared with WT-N group. Concentric remodeling was observed in WT-HFD, WT-LN, and WT-HFD-LN groups, but was highest in the WT-HFD-LN group. Concentric remodeling was shown by thicker LV walls (Fig. 1, *E* and *F*), as measured by conventional echocardiography (ECHO), and greater cardiomyocyte cross-sectional area (CSA) (Fig. 1, *M* and *O*). ECHO analysis did not show significant cardiac systolic or diastolic dysfunction in any group. LVEF (Fig. 1*G*) was preserved and LV longitudinal and radial strain and left ventricular internal diameter (Fig. 1*H*, Supplemental Fig. S2, *E* and *F*) were not significantly changed in all groups. The similar left atrium (LA) diameter (Fig. 1*I*) and *E*/*e*′ ratio (Fig. 1*J*) measured by ECHO, as well as similar left ventricular (LV) end-diastolic pressure (EDP) (Fig. 1*K*) and maximal rate of LV pressure decrease (dP/d*t*_min_) (Fig. 1*L*) from hemodynamic measurements indicated no significant diastolic dysfunction was present in WT-HFD, WT-LN, and WT-HFD-LN mice. Modest LV fibrosis was observed in the WT-LN and WT-HFD-LN groups (Fig. 1, *M* and *N*). WT-HFD-LN mice had the most cardiac remodeling compared versus other groups, but the lung weight (LuW) and gene expression level of atrial natriuretic peptide (ANP), two heart failure indicators, were not significantly increased (Supplemental Fig. S2, *D* and *G*). These data suggest that 4 mo of HFD plus l-NAME treatment induced some cardiac hypertrophy did not induce a robust HFpEF phenotype in FVB mice.

### Effects of High-Fat Diet and/or l-NAME on Mice with Low Levels of Expression of a Cardiac-Specific LTCC β2a-Subunit (3-Hit Mice)

**Figure 2.**
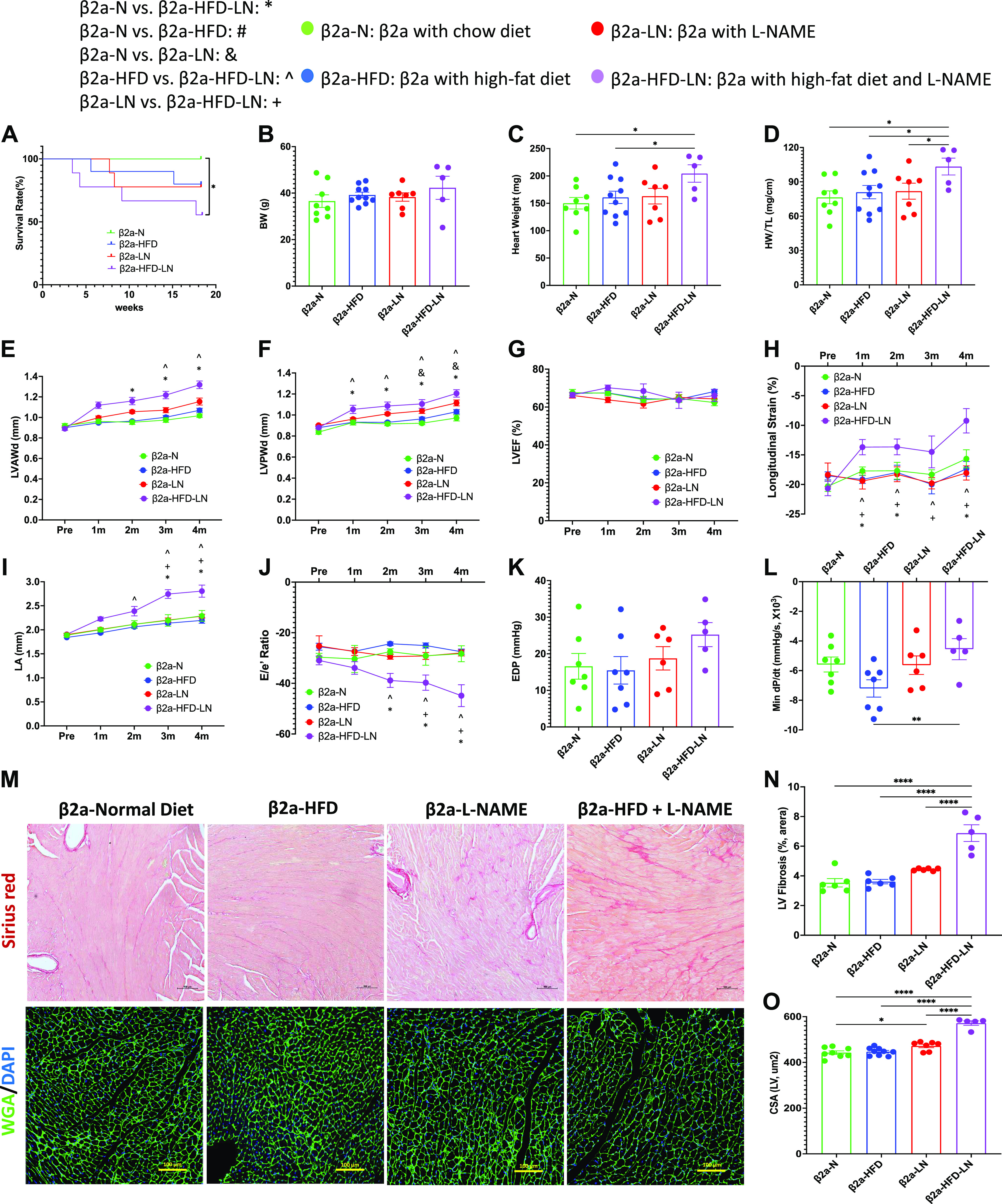
Effects of cardiac-specific L-type Ca^2+^ channel (LTCC) β2a-subunit expression together with high-fat diet (HFD) and *N*^ω^-nitro-l-arginine methyl ester (l-NAME, LN) treatment (3-Hit) on the heart failure with preserved ejection fraction (HFpEF) phenotype. *A*: survival rate from 4-mo follow-up. Body weight (BW; *B*), heart weight (HW; *C*), and ratio of HW to tibia length (HW/TL; *D*) at the time of euthanasia. Conventional and sophisticated echocardiography data showing left ventricular (LV) wall thickness (*E* and *F*), LV ejection fraction (LVEF; *G*), LV longitudinal strain (*H*), left atrium (LA) diameter (*I*), and ratio between early mitral inflow velocity (*E*), and mitral annular early diastolic velocity (*e′*) (*E*/*e*′; *J*). Hemodynamics data showing LV end-diastolic pressure (LVEDP; *K*) and maximum rate of pressure decay (dP/d*t*_min_; *L*). *M*: representative images of hearts stained with Picrosirius red and wheat germ agglutinin (WGA). *N*: quantification of the percentage of Picrosirius red-positive area. *O*: quantification of cardiomyocyte cross-sectional area (CSA). β2a, transgenic mouse with low levels of cardiomyocyte (CM)-specific inducible Cavβ2a expression; DAPI, 4′,6-diamidino-2-phenylindole; LVAWd: end-diastolic left ventricular anterior wall thicknesses; LVPWd, end-diastolic left ventricular posterior wall thickness; N, normal chow diet. Data shown are means ± SE. Tukey post hoc multiple comparison adjusted *P* values: *E–J*: *P* < 0.05, *β2a-N vs. β2a-HFD-LN, #β2a-N vs. β2a-HFD, &β2a-N vs. β2a-LN, ^β2a-HFD vs. β2a-HFD-LN, +β2a-LN vs. β2a-HFD-LN; and other panels: *P < 0.05, **P < 0.01, ****P < 0.0001. Total number of animals (*n*) and number of females and males included in each group are reported in the Supplemental Table.

Low levels of expression of cardiac-specific Cavβ2a-subunit were found in 4-mo-old mice, as previously reported (21) and remained constant through 8 mo of age (2.8-fold higher levels) (Supplemental Fig. S3, *A–C*). β2a-Tg mice were fed with a normal chow diet (β2a-N) or treated with a high-fat diet and/or l-NAME in water (β2a-HFD, β2a-LN, and β2a-HFD-LN; 3-Hit) for 4 mo (Supplemental Fig. S1). HFD treatment showed a trend to increase the mean BW (Fig. 2*B*), while l-NAME treatment increased the blood pressure of β2a mice (Supplemental Fig. S3, *D* and *E*).

The data from four β2a groups (β2a-N, β2a-HFD, β2a-LN, and β2a-HFD-LN; 3-Hit) were compared (see Fig. 2). β2a-HFD-LN (3-Hit) mice had premature mortality compared with β2a-N mice (Fig. 2*A*), and a more severe cardiac phenotype compared with β2a-N, β2a-HFD, and β2a-LN mice. Cardiac hypertrophy in β2a-HFD-LN mice was shown by a greater HW, HW/TL (Fig. 2, *C* and *D*), thicker LV walls (Fig. 2, *E* and *F*), and a greater cardiomyocyte cross-sectional area (CSA) (Fig. 2, *M* and *O*) versus other groups. ECHO analysis showed all four groups of mice had preserved LVEF (>50%) (Fig. 2*G*), but β2a-HFD-LN mice had significant decreases in LV longitudinal (<16%) strain (Fig. 2*H*), suggesting some impairment of systolic function. ECHO and hemodynamic measurements both showed a more significant cardiac diastolic dysfunction in β2a-HFD-LN mice than in any other group. There was a significant increase in left atrium (LA) diameter and *E*/*e*′ ratio (Fig. 2, *I* and *J*) measured by ECHO, and an increased EDP and decreased dP/d*t*_min_ determined by invasive hemodynamics (Fig. 2, *K* and *L*) in β2a-HFD-LN mice. In addition, LV fibrosis was most severe in the β2a-HFD-LN group (Fig. 2, *M* and *N*). These data document that 3-Hit mice had the most severe pathological phenotype of the mice studied.

### The 3-Hit Mouse Model Produces a Profound HFpEF Phenotype, Which Can Be Reduced by SAHA Treatment

To clarify the similarities and differences between WT and β2a-Tg mice after treatment with HFD and LN, and the effect of SAHA [suberoylanilide hydroxamic acid, vorinostat, a pan-HDAC activity inhibitor (32, 33)] treatment, the data from five groups (WT-N, WT-HFD-LN, β2a-N, β2a-HFD-LN, and β2a-HFD-LN-SAHA) were compared.

**Figure 3.**
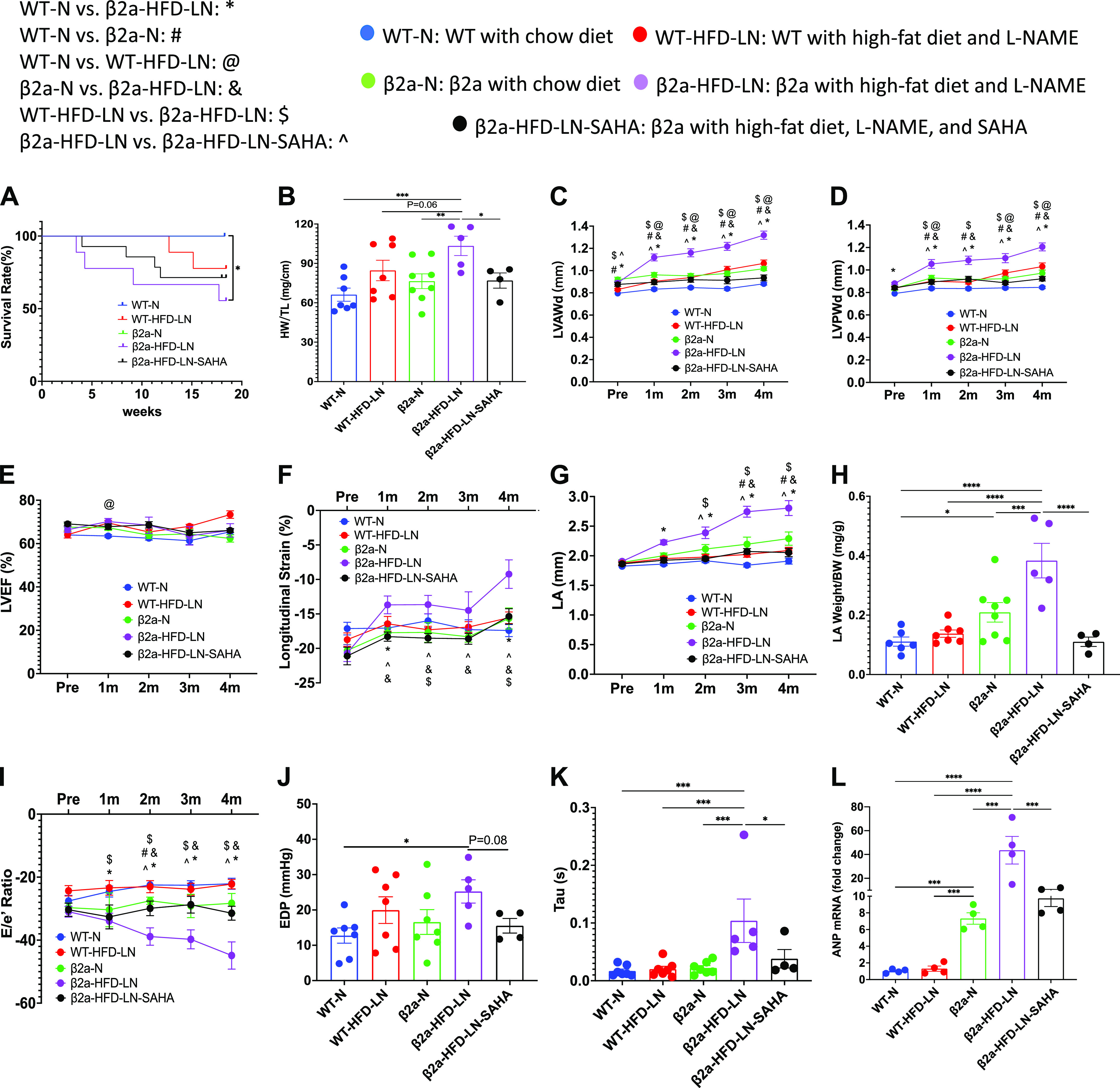
The 3-Hit mouse model produces a profound heart failure with preserved ejection fraction (HFpEF) phenotype, which can be reduced by suberoylanilide hydroxamic acid (SAHA) treatment. β2a, transgenic mouse with low levels of cardiomyocyte (CM)-specific inducible Cavβ2a expression; HFD, high-fat diet; l-NAME (or LN), *N*^ω^-nitro-l-arginine methyl ester; N, normal diet; WT, wild type. Data shown for WT-N, WT-HFD-LN, β2a-N, and β2a-HFD-LN groups in are the same as those shown in Figs. 1 and 2, and Supplemental Fig. S2. Statistical comparisons being made here are unique. The β2a-HFD-LN-SAHA group was added into the statistic comparation. *A*: survival rate from 4-mo follow-up. *B*: body weight (BW) at the time of euthanasia. Conventional and sophisticated echocardiography data showing left ventricular (LV) wall thickness (*C* and *D*), LV ejection fraction (LVEF; *E*), LV longitudinal strain (*F*), and left atrium (LA) diameter (*G*). *H*: LA weight-to-BW ratio at the time of euthanasia. *I*: conventional echocardiography data showing ratio between early mitral inflow velocity (*E*) and mitral annular early diastolic velocity (*e′*) (*E*/*e*′). Hemodynamics data showing end-diastolic pressure (EDP; *J*) and LV diastolic time constant (τ; *K*). *L*: expression level of atrial natriuretic peptide (ANP) in heart tissues by real-time polymerase chain reaction. Relative expression was calculated with respect to the WT-N group. HW, heart weight; LVAWd, end-diastolic left ventricular anterior wall thicknesses; LVPWd, end-diastolic left ventricular posterior wall thickness; TL, tibia length. Data shown are means ± SE. Tukey post hoc multiple comparison adjusted *P* values: *C–G* and *I*: *P* < 0.05, *WT-N vs. β2a-HFD-LN, #WT-N vs. β2a-N,@WT-N vs. WT-HFD-LN, &β2a-N vs. β2a-HFD-LN, and $WT-HFD-LN vs. β2a-HFD-LN; and other panels: *P < 0.05, **P < 0.01, ***P < 0.001, ****P < 0.0001. Total number of animals (*n*) and number of females and males included in each group are reported in the Supplemental Table.

The death rate during the 4-mo study was significantly greater in β2a-HFD-LN animals compared with WT-N (Fig. 3*A*). The BW and blood pressure (systolic and diastolic pressure) were significantly greater in β2a-HFD-LN mice when compared with WT-N mice (Supplemental Fig. S4, *A–C*). More severe cardiac hypertrophy in β2a-HFD-LN mice was shown by significantly increased HW, HW/TL, HW/BW, and LV wall thickness (Supplemental Fig. S4, *D* and *E*; Fig. 3, *B*–*D*) versus other groups. In addition, the β2a-Tg mice had thicker LV walls at 4 mo of age compared with WT mice (Fig. 3, *C* and *D*), which was consistent with our previous findings (21).

ECHO analysis showed β2a-HFD-LN mice had preserved LVEF (>50%) (Fig. 3*E*), but significantly decreased LV longitudinal strain (<16%) (Fig. 3*F*). Histological, ECHO, and hemodynamic measurements showed significant cardiac diastolic dysfunction. The evidence included a significant increase in left atrium (LA) diameter (Fig. 3*G*), LA weight/BW (Fig. 3*H*), *E*/*e*′ ratio (Fig. 3*I*), and increased EDP (Fig. 3*J*), left ventricular diastolic time constant (τ) (Fig. 3*K*), and decreased dP/d*t*_min_ (Supplemental Fig. S4*G*). When compared with other groups, ANP gene expression was significantly greater in β2a-HFD-LN mice (Fig. 3*L*). These results show that the combination of three stressors, cardiac-specific β2a-subunit plus HFD plus l-NAME treatment (3-Hit) induces a profound HFpEF phenotype that could cause premature death.

SAHA treatment groups did not show significant decreases in survival rate compared with WT-N group (Fig. 3*A*). In addition, SAHA treatment caused a significant decrease in HW, HW/BW, HW/TL, and LV wall thickness versus β2a-HFD-LN mice (Fig. 3, *B–D* and Supplemental Fig. S4, *D* and *E*). SAHA treatment did not affect LVEF (Fig. 3*E*) but prevented the decrease in longitudinal (Fig. 3*F*) in β2a-HFD-LN mice. ECHO measurements documented significantly decreased LA diameter, LA weight/BW, and absolute *E*/*e*′ ratio (Fig. 3, *G*–*I*) in β2a-HFD-LN-SAHA mice indicating improved LV diastolic function. Lower EDP and τ in SAHA-treated mice was observed (Fig. 3, *J* and *K*). SAHA treatment also caused a significant decrease in ANP expression (Fig. 3*L*). These data show that SAHA treatment prevented early stage systolic dysfunction and diastolic dysfunction in the 3-Hit mice.

### The 3-Hit Mouse Has More Cardiomyocyte Hypertrophy and Necrosis, and SAHA Treatment Can Alleviate the Phenotype

**Figure 4.**
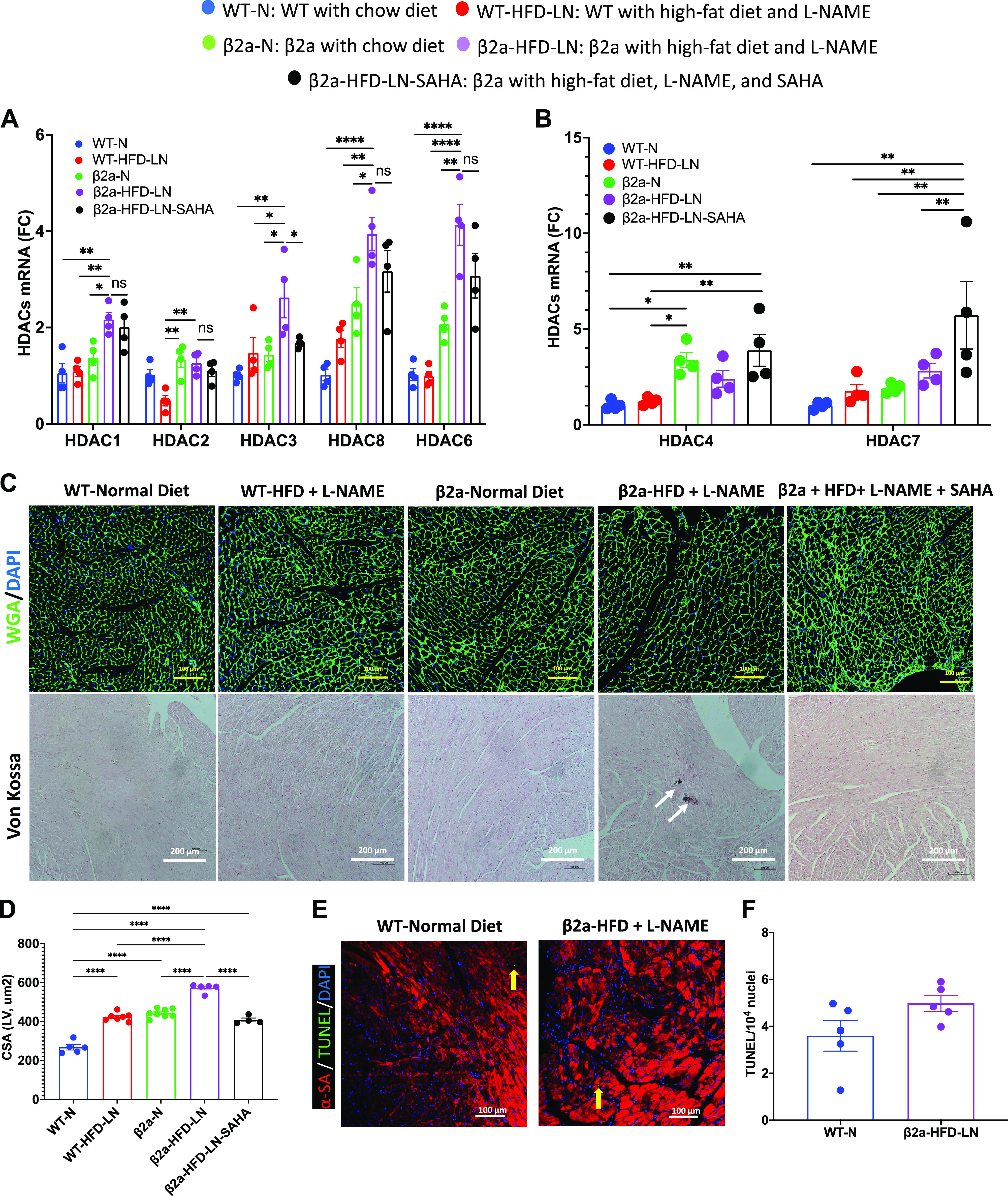
The 3-Hit mouse model has a more severe cardiomyocyte hypertrophy and necrosis, which can be reduced by suberoylanilide hydroxamic acid (SAHA) treatment. β2a, transgenic mouse with low levels of cardiomyocyte (CM)-specific inducible Cavβ2a expression; HFD, high-fat diet; N, normal chow diet, l-NAME (LN), *N*^ω^-nitro-l-arginine methyl ester; WT, wild type. Representative images of wheat germ agglutinin (WGA)-stained hearts (*C*) and quantification of cardiomyocyte cross-sectional area (CSA) data (*D*) in WT-N, WT-HFD-LN, β2a-N, and β2a-HFD-LN groups are the same as those shown in Fig. 1, *M* and *O*, and Fig. 2, *M* and *O*. The statistical comparisons being made here are different than those made in Figs. 1 and 2. The β2a-HFD-LN-SAHA group was added into the statistic comparison. *A* and *B*: expression level of class I histone deacetylases (HDACs) 1, 2, 3 and 8, Class IIb HDAC6 (*A*) and class IIa HDACs 4 and 7 (*B*) in heart tissues by real-time polymerase chain reaction. Relative expression was calculated with respect to WT-N mice compared with different treatment groups. *C*: representative images of WGA-stained hearts and histological assessment of cardiac ventricular pathology by Von Kossa staining. *D*: quantification of cardiomyocyte CSA. *E* and *F*: representative images of terminal deoxynucleotidyl transferase dUTP nick end labeling (TUNEL)-stained hearts (*E*) and quantification of TUNEL-positive myocyte nuclei from hearts (*F*): α-sarcomeric actin (α-SA), red; TUNEL, green; and 4′,6-diamidino-2-phenylindole (DAPI), blue. Data shown are means ± SE. Tukey post hoc multiple comparison adjusted *P* values are reported here. *P < 0.05, **P < 0.01, ****P < 0.0001.

HDACs are known to be centrally involved in pathological cardiac hypertrophy (34, 35). Activation of Class I HDACs (HDACs 1, 2, 3, and 8) and Class IIb HDACs (HDAC6) are thought to promote pathological hypertrophy, whereas class II HDACs (HDACs 4, 5, 7, and 9) are thought to suppress cardiac hypertrophy. In WT-N and β2a-HFD-LN mice, the most abundant HDACs (Class I and Class IIb) expressed in the heart were HDACs 1, 2, 3, 8, 4, and 7 (Supplemental Fig. S5). HDAC1, 3, 6, and 8 were significantly higher in β2a-HFD-LN mice compared with WT-N, WT-HFD-LN, and β2a-N groups (Fig. 4*A*). SAHA treatment group showed a significant decrease in HDAC3 expression, and an increase in HDAC4 and 7 (Fig. 4, *A* and *B*).

β2a-HFD-LN, 3-Hit mice had the greatest increase in myocyte CSA compared with WT-N, and WT-HFD-LN, indicating the most severe myocyte hypertrophy (Fig. 4, *C* and *D*). The CM size in β2a-HFD-LN-SAHA group was smaller (Fig. 4, *C* and *D*) than in β2a-HFD-LN mice. There was no significant difference in the number of myocytes undergoing apoptosis (TUNEL staining) between WT-N and β2a-HFD-LN mice (Fig. 4, *E* and *F*). In contrast, Von Kossa staining showed that only β2a-HFD-LN mice had evidence of more Ca^2+^ deposition, suggestive of CM necrosis (22) (Fig. 4*C*). Collectively, these results show that SAHA treatment reduced cardiomyocyte hypertrophy and necrosis caused by HFD plus l-NAME treatment in β2a-Tg mice.

### The 3-Hit Mouse Has Increased Cardiac M_2_-Macrophages and Fibroblast Activation, Which is Reduced by SAHA Treatment

**Figure 5.**
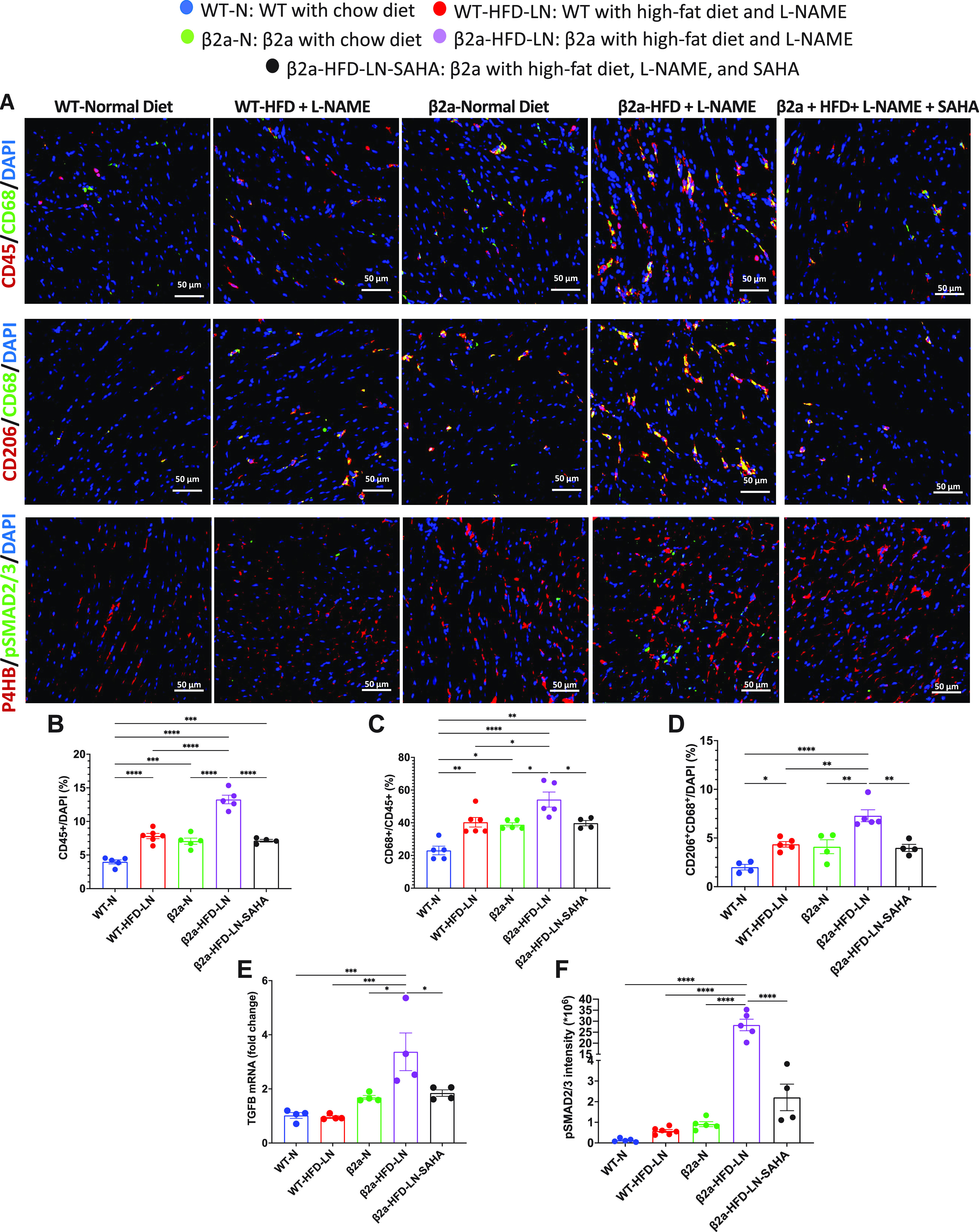
The 3-Hit model has robust cardiac M_2_-macrophage infiltration and fibroblast activation, which can be reduced by suberoylanilide hydroxamic acid (SAHA) treatment. *A*: immunofluorescence staining of heart sections to show CD45^+^ (red) immune cell, CD45^+^ (red) CD68^+^ (green) macrophage, CD68^+^ (green) CD206^+^ (red) M_2_-macrophage, and phosphorylated Smad2/3 (pSmad2/3, green) in fibroblast (red). 4′,6-Diamidino-2-phenylindole (DAPI) was stained as blue. Data are expressed as percentage of CD45^+^ cell/total cells (*B*), percentage of CD68^+^ cell/CD45^+^ cell (*C*), and CD206^+^CD68^+^/total cell (*D*). *E*: expression level of transforming growth factor-β (TGFβ) in heart tissues by real-time polymerase chain reaction. Relative expression was calculated with respect to wild-type/normal chow diet (WT-N) mice compared with different treatment groups. *F*: quantification of mean intensity of pSmad2/3 for each group. β2a, transgenic mouse with low levels of cardiomyocyte (CM)-specific inducible Cavβ2a expression; HFD, high-fat diet; l-NAME (LN), *N*^ω^-nitro-l-arginine methyl ester; P4HB, protein disulfide isomerase/prolyl 4-hydroxylase. Data shown are means ± SE. Tukey post hoc multiple comparison adjusted *P* values are reported here. *P < 0.05, **P < 0.01, ***P < 0.001, ****P < 0.0001.

Cardiac hypertrophy and necrosis are associated with interstitial cardiac fibrosis in HFpEF (36, 37). We counted CD45^+^, CD68^+^, and CD206^+^ cells, to estimate the number of macrophages, and M_2_-macrophages, respectively. Significantly more CD45^+^ monocytes were present in β2a-HFD-LN hearts than in other groups (Fig. 5, *A* and *B*). The β2a-HFD-LN mice also had a significantly higher ratio of CD68^+^/CD45^+^ macrophages, and more CD206^+^ CD68^+^ M_2_-like macrophage than in hearts from other groups (Fig. 5, *C* and *D*). SAHA treated β2a-HFD-LN hearts had fewer CD45^+^ monocytes, CD68^+^/CD45^+^ macrophages, and CD206^+^CD68^+^ profibrotic M_2_-macrophage population than in untreated β2a-HFD-LN hearts (Fig. 5, *A*–*D*).

Macrophages are a potent transforming growth factor-β (TGFβ) producer (38). TGFβ mRNA was significantly increased in β2a-HFD-LN hearts (Fig. 5*E*). The phosphorylation levels of Smad2 and 3 (pSmad2/3), two major molecules known to be downstream of TGFβ signaling, were also increased in β2a-HFD-LN hearts (Fig. 5*F*). The mRNA level of TGFβ and pSmad2/3 were significantly lower in β2a-HFD-LN-SAHA mice compared with β2a-HFD-LN mice (Fig. 5, *E* and *F*). These results support the idea that in this 3-Hit HFpEF model, profibrotic M_2_-macrophages activate TGFβ signaling to stimulate cardiac fibroblast activation (differentiation into myofibroblasts) to promote fibrosis, which was reduced by SAHA treatment.

### SAHA Treatment Reduces Myocardial Fibrosis in the 3-Hit Mouse

**Figure 6.**
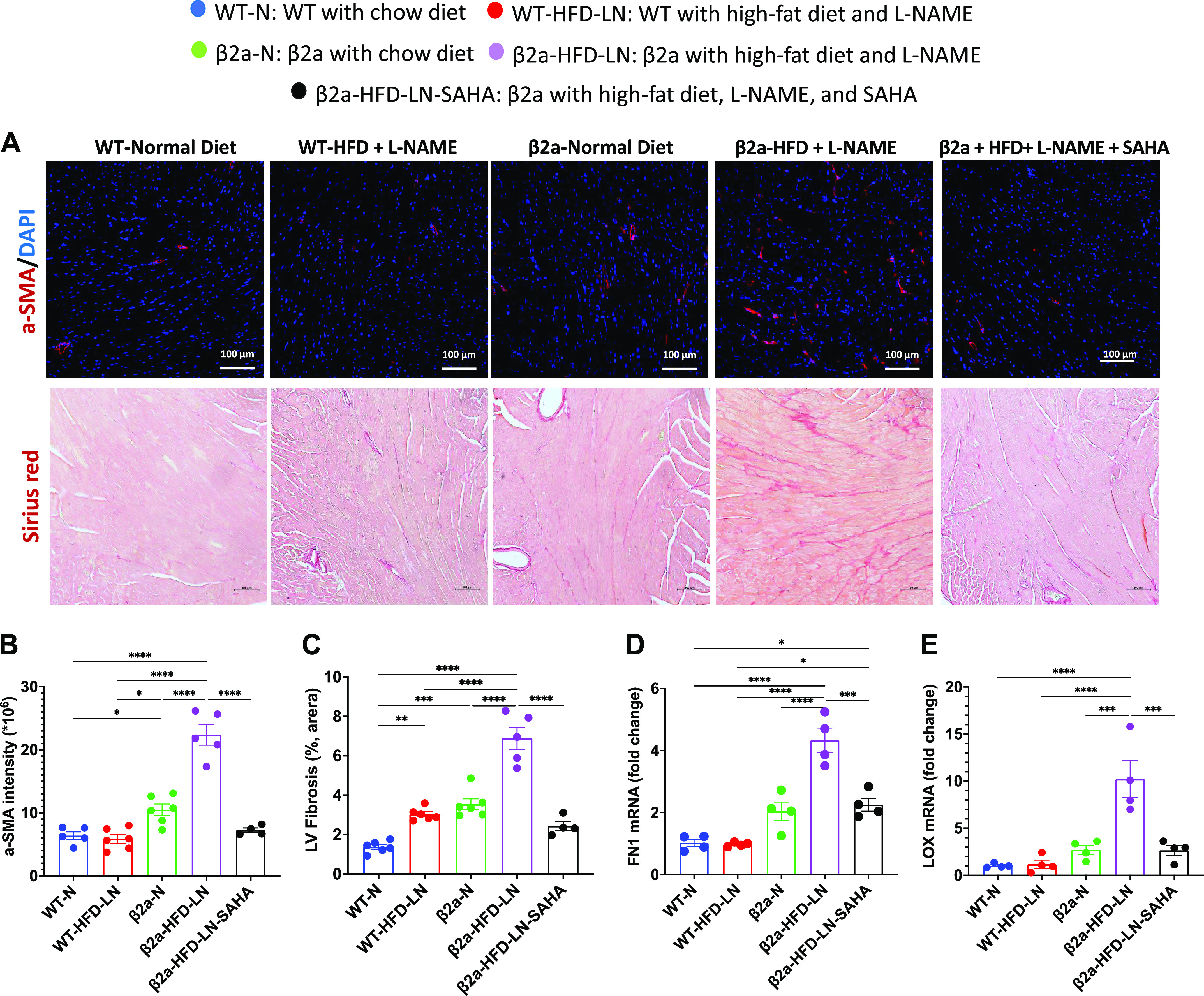
The 3-Hit model has severe myocardial fibrosis, which can be reduced be suberoylanilide hydroxamic acid (SAHA) treatment. β2a, transgenic mouse with low levels of cardiomyocyte (CM)-specific inducible Cavβ2a expression; HFD, high-fat diet; N, normal chow diet, l-NAME (LN), *N*^ω^-nitro-l-arginine methyl ester; WT, wild type. The representative images of Picrosirius red-stained hearts (*A*) and quantification of the percentage of Picrosirius red-positive area (*C*) data of WT-N, WT-HFD-LN, β2a-N, and β2a-HFD-LN groups are the same as in Fig. 1, *M* and *N*, and Fig. 2, *M* and *N*. The statistical comparisons being made are different than those made in Figs. 1 and 2. The β2a-HFD-LN-SAHA group was added into the statistic comparation. *A*: representative images of hearts from 4 groups with α-smooth muscle actin (α-SMA) (red) immunofluorescence staining and Picrosirius red staining. Quantification of the α-SMA (red) intensity (*B*) and the percentage of Picrosirius red-positive area (*C*). Expression level of fibronectin 1 (FN1; *D*) and collagen cross-linking enzyme lysyl oxidase (LOX; *E*) in heart tissues by real-time polymerase chain reaction. Relative expression was calculated with respect to WT-N mice compared with different treatment groups. DAPI: 4′,6-diamidino-2-phenylindole. Data shown are means ± SE. Tukey post hoc multiple comparison adjusted *P* values are reported here. *P < 0.05, **P < 0.01, ***P < 0.001, ****P < 0.0001.

The expression of α-smooth muscle actin [α-SMA, a biomarker for activated myofibroblast (39, 40)], collagen, and fibronectin 1 (FN1), are upregulated by the transcription factors pSmad2/3 (41). β2a-HFD-LN hearts had a higher α-SMA staining intensity (immunofluorescence) and more interstitial fibrosis (Picrosirius red staining) (Fig. 6, *A*–*C*) than found in other groups. In addition, the mRNA levels of FN1 and collagen cross-linking enzyme lysyl oxidase (LOX) were increased in β2a-HFD-LN hearts (Fig. 6, *D* and *E*). After SAHA treatment, α-SMA intensity was reduced, as were cardiac interstitial fibrosis, and mRNA level of FN1 and LOX when compared with β2a-HFD-LN mice (Fig. 6, *A*–*E*). Expression levels of matrix metalloproteinase-9 (MMP9), a proteinase involved in fibrosis (42), were not different among groups (Supplemental Fig. 4*H*).

These results suggest that the combination of three stressors in β2a-HFD-LN hearts produces severe cardiac hypertrophy and cell necrosis with increased profibrotic M_2_-macrophage populations, TGFβ-dependent cardiac fibroblast activation, and myocardial fibrosis, ultimately leading to a profibrotic HFpEF phenotype. SAHA treatment prevents these HFpEF features.

## DISCUSSION

Heart failure with preserved ejection fraction (HFpEF) is a major public health problem with an increasing prevalence (3–6), high morbidity and mortality (7), and a high rehospitalization rates (8). HFpEF treatments are not well established (1) and new therapies need to be developed. Animal models that have critical features of human HFpEF pathophysiology could help define the cellular and molecular bases of HFpEF induction and progression and identify targets for new therapies. This study explored the characteristics of a new 3-Hit mouse model [cardiomyocyte-specific increases in Ca^2+^ influx, plus high-fat diet (HFD) plus l-NAME] to determine if it has crucial phenotypic features of HFpEF.

HFpEF is a complex syndrome that has many causes in humans and the 3-Hit model characterized in this study was developed with the idea that more than one pathological stressor may be needed to induce a profound phenotype reminiscent of human HFpEF. Our 3-Hit model combines cardiac myocyte-specific Ca^2+^ stress, systemic metabolic stress, and vascular stress. Our results show that each of these three stressors alone induce modest cardiac phenotypes but do not cause a profound HFpEF phenotype. Combining HFD and l-NAME in WT mice did not cause a profound HFpEF phenotype, as also shown in a recent study (17). Likewise, combining Ca^2+^ stress with one of the other two stressors also did not result in a profound phenotype. Only the combination of these three stressors was able to induce a profound HFpEF phenotype with premature death.

We identified a mechanistic role for HDAC dependent-CM hypertrophy and necrosis that was associated with increased profibrotic M_2_-macrophage population, fibroblast activation, and myocardial fibrosis. Finally, we showed that treatment of β2a-HFD-LN mice with SAHA (a pan-HDAC inhibitor) prevented the severe HFpEF phenotype and the associated premature death.

### l-NAME Can Cause Hypertension and Cardiac Remodeling but Alone Does Not Induce a Profound HFpEF Phenotype

Hypertension, and vascular dysfunction can cause cardiac remodeling that includes myocyte hypertrophy and fibrosis (43). Chronic administration of the nitric oxide (NO) synthase inhibitor l-NAME is known to causes arterial hypertension in mouse model (44). In the present experiments, treatment of WT mice with l-NAME caused hypertension but only induced modest changes in cardiac structure and function. These changes were not sufficient to cause a profound HFpEF phenotype (Fig. 1).

β2a-Tg mice have a basal hypertrophic phenotype with modest fibrosis (see discussion later). Treatment with l-NAME caused an increase in blood pressure but did not induce a profound exacerbation of the β2a phenotype (Fig. 2). These results show that the effects of l-NAME contribute to a cardiovascular phenotype, but at least with the conditions we employed, a pronounced phenotype was not observed when the effects of l-NAME alone were studied, and the phenotype was not substantially increased when l-NAME treatment was combined with HFD (in WT mice, Fig. 1) or β2a stress (Fig. 2).

### High-Fat Diet Causes Cardiac Remodeling but Alone Does Not Induce a Profound HFpEF Phenotype

HFD induces weight gain and metabolic disturbances in mice (45) and in humans (46). The obesity epidemic in Western society is now clearly linked to the development of HFpEF in younger and younger patients (47, 48). In the present study, HFD in WT mice caused modest cardiac hypertrophy and fibrosis but the overall phenotype was mild (Fig. 1). Adding l-NAME to WT mice fed a HFD induced an increase in blood pressure but did not substantially increase the cardiac remodeling response (Fig. 1; Supplemental Fig. S2). Feeding β2a-Tg mice a HFD caused them to gain weight but did not induce a significant exacerbation of the β2a phenotype and did not cause profound HFpEF signs and symptoms (Fig. 2).

A recent study from Dr. Hill’s laboratory (17) that the combination of two stressors (HFD and l-NAME) in C57BL/6 mice induced features of human HFpEF. Their results showed that with HFD + l-NAME treatment, WT mice already had increased BW, blood pressure, HW/TL, lung weight, cardiac ANP expression, cardiomyocyte CSA, and myocardial fibrosis. In addition, this 2-Hit mouse model showed preserved LVEF but decreased LV longitudinal strain, as well as a significantly altered LV diastolic function evaluated by both ECHO and hemodynamic.

In our study, we treated WT FVB mice with HFD and l-NAME for 4 mo. We observed some HFpEF features, including increased BW, blood pressure, preserved LVEF, increased cardiomyocyte CSA, and myocardial fibrosis (Fig. 1; Supplemental Fig. S2). However, we failed to observe a significantly increased HW/TL ratio, lung weight/TL ratio, and cardiac ANP expression (Fig. 1; Supplemental Fig. S2). In addition, the WT-HFD-LN groups did not show significantly decreased LV longitudinal and radial strain and LV diastolic dysfunction measured by both ECHO and hemodynamic (Fig. 1; Supplemental Fig. S2). These results suggest that FVB mice do not develop critical HFpEF features with the HFD + l-NAME.

The potential reasons for our results likely involve the distinct genetic backgrounds of FVB and C57BL/6 mice, which have been reported to have an influence on mouse baseline cardiac function and the response to different stimuli (49). In addition, FVB and C57BL/6 mouse strains have different behaviors in relation to metabolism (50). The C57BL/6 mouse also has a more robust effect on the innate immune system than seen in FVB mice after HFD (50). The FVB mouse is considered diet‐resistant (51) and has low HFD-induced atherosclerosis susceptibility (52).

### Ca^2+^ Stress Can Induce Cardiac Hypertrophy without a Profound HFpEF Phenotype

It is well established that many cardiac diseases increase the work demands of cardiac myocytes (53, 54). Increases in Ca^2+^ influx enhances contraction to meet increased physiological and pathological demands (21, 54–56). The increased cellular Ca^2+^ causes increased contraction but also elicits a host of responses including cardiac hypertrophy (21, 54–56), metabolic reprogramming (57), and can lead to cell death (22, 58). There is a robust literature linking increased Ca^2+^ influx in disease to cardiac remodeling (21, 54–56, 59).

We used a genetically modified mouse with low levels of cardiac myocyte-specific expression of the β2a-subunit of the l-type Ca^2+^channel (21). The present experiments show that low levels of β2a expression can induce a hypertrophic phenotype (Figs. 3 and 4). The Ca^2+^ disturbances induced in this mouse could be similar to those that are present in a variety of mild disease states. We choose this mouse model because it induced low levels of Ca^2+^ stress without a severe cell death phenotype (22). Our new results show that adding HFD alone or l-NAME alone to β2a-Tg animals did not induce a severe exacerbation of the β2a phenotype (Fig. 2).

Our results show that any two combinations of Ca^2+^ stress, HFD, and l-NAME were not sufficient to cause a profound HFpEF phenotype with significant premature death. Why this occurs is not entirely clear since all three stressors cause some degree of cardiac hypertrophy and fibrosis. Our experiments show that when these three independent stressors were applied to FVB mice together, they collectively induced a severe HFpEF phenotype associated with significant levels of premature death (Figs. 3 and 4).

### Three-Hit Mouse Model Meets All Criteria Necessary for HFA-PEFF HFpEF Diagnosis

The clinical complexity of HFpEF and the lack of a single objective marker make diagnosing of HFpEF difficult. Recently, HFA-PEFF (60) clinical algorithms have been developed to improve and standardize the diagnosis of HFpEF. HFA-PEFF diagnostic algorithms, proposed by the Heart Failure Association (HFA) of the European Society of Cardiology, is a stepwise diagnostic algorithm, which can be easily and accurately calculated, and is useful for predicting composite cardiovascular events as well as HF-related events in patients with HFpEF (61–63). It assesses the pretest probability of HFpEF based on clinical features (including age and comorbidities) and cardiac functional and structural echocardiographic data, including morphological aspects of the LA and LV, as well as levels of natriuretic peptides (60). Several different HFpEF preclinical models had been scored using HFA-PEFF (18). Our 3-Hit mouse model showed comorbidity burden of heart failure (obesity and increased blood pressure), decreased global longitudinal strain (<16%), diastolic dysfunction (increased absolute *E*/*e*′, ≥15), enlarged LA, increased heart weight, thicker LV wall, LV hypertrophy, and increased natriuretic peptides expression in heart tissue (Figs. 2, 3, and 4). These results show that the 3-Hit model meet the criteria necessary for an HFA-PEFF HFpEF diagnosis with a high score. In addition, while our 3-Hit model had preserved LVEF, we found a decreased LV longitudinal and radial strain. Strain is an ECHO parameter widely used in clinical work to detect mild cardiac systolic impairment at an early stage before LVEF decreases. Several clinical studies have already shown that patients with HFpEF are characterized by decreased LV strain (64, 65), and our 3-Hit model also mirrors the mild cardiac systolic impairment phenotype shown in these studies.

### 3-Hit Mouse Model Involves HDAC Activation

Cardiac hypertrophy is one of the best-studied aspects of HFpEF and is a common clinical feature of HFpEF (9). Patients with HFpEF tend to have normal LV filling volumes, with variable degrees of LV wall thickening (66–68). Gupta et al. (68) showed that ∼75% of patients with HFpEF had significant cardiac hypertrophy. Cardiac hypertrophy is also associated with the diastolic dysfunction and elevated diastolic filling pressure observed in HFpEF (9, 69). Pathophysiological cardiac hypertrophy in patients with HFpEF likely involves impaired Ca^2+^ handling (9), myocardial fibrosis (4, 9), oxidative stress (9, 70), cell death (9, 22, 58), metabolic reprogramming (57), and induction of fetal genes (9, 71). All of these features are induced in the 3-Hit mouse model characterized in the present work.

One of the known molecular contributors to pathological cardiac hypertrophy is the activation of HDACs (72, 73), which remove *N*-acetyl-lysine from histone and non-histone proteins to induce cardiac hypertrophy, fibrosis, and diastolic dysfunction (35, 74–76). HDACs fall into four distinct classes (I, II, III, and IV). Class I HDACs (HDACs 1, 2, 3, and 8) and Class IIb HDAC (HDAC6) promote pathological hypertrophy, whereas Class IIa HDACs (HDACs 4, 5, 7, and 9) suppress cardiac hypertrophy (35, 77). Experiments with the 3-Hit mouse model show that they had severe cardiac hypertrophy, diastolic dysfunction, and a profound inflammatory and fibrotic response (Figs. 3, 4, and 5, Supplemental Fig. S4). Significant changes in the expression ratio of different HDACs were observed in heart tissue of WT-N versus β2a-HFD-LN hearts. The main class I and class IIa HDACs expressed in all the hearts were HDACs 1, 2, 3, 8, 4, and 7 (Supplemental Fig. S5). Cardiac hypertrophy-inducing HDACs (HDAC1, 3, 6 and 8) were significantly increased in β2a-HFD-LN hearts (Fig. 4). The role of specific HDAC isoforms will require further study and could lead to novel approaches to abrogate the HFpEF phenotype in the 3-Hit model.

A role for HDAC activation in the HFpEF phenotype of β2a-HFD-LN was further documented in studies with the pan-HDAC inhibitor SAHA, an agent capable of promoting regression of established hypertrophy (32, 33). Our experiments showed that SAHA prevented the development of a severe HFpEF phenotype in 3-Hit mice. SAHA also reduced the necrosis, inflammation, and fibrosis that were shown to be responsible for the development of the profound HFpEF phenotype seen in the 3-Hit model (Figs. 3, 4, 5, and 6; Supplemental Fig. S6).

### Increased Profibrotic M_2_-macrophage-TGFβ-Fibroblast Activation-Myocardial Fibrosis Pathway in 3-Hit Mouse Model Mirror the Clinical HFpEF Pathological Features

Obesity, hypertension, and cardiac hypertrophy have long been considered to create a profibrotic environment, stimulating the interstitial cardiac fibrosis that contributes to passive muscle stiffening and reduced chamber compliance in HFpEF (36, 37). One of the known signaling mechanisms that can contribute to cardiac fibrosis is TGFβ. Macrophages are a potent producer of TGFβ. Macrophages have two main phenotypes: the M1 (classically activated) and M_2_ (alternatively activated and profibrotic)-macrophages. Westermann et al. (78) reported an increased number of TGFβ-expressing leukocytes, features characteristic of M_2_-macrophages, in HFpEF cardiac biopsies. Glezeva et al. (79) reported similar results that increased peripheral inflammation, monocytosis, and monocyte differentiation to anti-inflammatory/profibrotic M_2_-macrophages were present in HFpEF. The role of M_2_-macrophage has not been well studied in HFpEF animal models. In one recently published study, uninephrectomy and d-aldosterone infusion were performed to create a HFpEF mouse model. This study reported decreased mRNA expression of M_2_ markers (Arg1, CD163, and CD206) in HFpEF hearts, which was opposite from clinical patients with HFpEF (80). Our results show that the number of M_2_-macrophages was significantly increased in the 3-Hit mouse with a significant HFpEF phenotype (Fig. 5). More work is needed to more clearly define the role of TGFβ signaling and profibrotic inflammatory processes in HFpEF.

TGFβ, secreted by macrophages, binds to the TGF receptor and actives a cascade of intracellular signals through the phosphorylation of Smad2 and -3 (41). The pSmad2/3 translocate into the nucleus and binds to transcription factors (Smad binding element, SBE) on DNA, and then regulates the downstream gene expression, including α-SMA, collagen fiber, FN1, etc. (81). The activation of TGFβ dependent Smad2/3 pathway in cardiac fibroblast cells is thought to contribute to the development of fibrosis, where fibroblasts transform into myofibroblast cells (40). The crosslinking of extracellular matrix proteins regulated by lysyl oxidase (LOX) also potently affects their mechanical properties (82). In our study, the 3-Hit mouse model developed severe CM hypertrophy with some cell necrosis. These changes were associated with increased cardiac profibrotic M_2_-macrophage population, TGFβ secretion, phosphorylation of Smad2 and -3, fibroblast activation, expression of FN1 and LOX, and myocardial fibrosis (Figs. 5 and 6). These pathological changes were linked to increased myocardial stiffness and LV filling pressures that underlie diastolic dysfunction in the 3-Hit model.

HDACs inhibitors have been shown to reduce collagen production and decrease markers of cardiac fibrosis in the diseased heart (83, 84). The present study showed the importance of these HFpEF mechanisms in SAHA treatment experiments. These experiments showed that SAHA treatment of 3-Hit mice can prevent the development of severe cardiac hypertrophy, increased M_2_-macrophage population, TGFβ secretion, fibroblast activation, and myocardial fibrosis (Figs. 4, 5, and 6). Collectively, these data suggest that the 3-Hit model could be used to further define HFpEF mechanisms and for testing putative new therapies.

### Limitations

HFpEF is a complex clinical syndrome and is increasingly being recognized as a multiorgan, systemic syndrome. HFpEF animal models with one inducing stressor are, therefore, likely to have limitations that make cellular and molecular mechanisms that characterize this syndrome less well understood. Our goal was to develop a preclinical HFpEF animal model with multiple stressors known to be linked to HFpEF that together induce a profound HFpEF phenotype.

Our 3-Hit HFpEF mouse model with cardiomyocyte-specific Ca^2+^ stress plus HFD and l-NAME treatment mirrored the HFpEF clinical phenotype. A limitation of the current study is the small size of some of the treatment groups and the fact that sex-based effects in the model still need to be determined (85). Another limitation of the study is the relatively long treatment period, which made the model building more challenging. The mice were 8 mo old when studies were terminated, so aging could be a factor contributing to the results. In addition, our study did not include the exercise exhaustion test, which can detect the exercise intolerance commonly present in patients with HFpEF. An advantage of the current study is that the β2a-N mice had a lower β2a expression and a less pronounced phenotype (22) that allowed us to explore the added stress of HFD and l-NAME.

### Conclusions

In summary, the 3-Hit mouse model produced a profound HFpEF phenotype. The primary mechanisms inducing this phenotype were HDACs dependent-CM hypertrophy, necrosis, increased profibrotic M_2_-macrophage populations, fibroblast activation, and myocardial fibrosis. A role for HDAC activation in the HFpEF phenotype was shown in studies with SAHA treatment, which prevented the severe HFpEF phenotype. These results suggest that this 3-Hit mouse model can be used for identifying and testing novel therapeutic strategies to treat HFpEF.

## DATA AVAILABILITY

Data will be made available upon reasonable request.

## SUPPLEMENTAL DATA

10.6084/m9.figshare.22068815 Supplemental Table S1 and Figs. S1–S5: https://www.doi.org/10.6084/m9.figshare.22068815.

## GRANTS

This study was funded by National Heart, Lung, and Blood Institute Grants HL140071 (to S. R. Houser) and HL147558 (to S. R. Houser and T. A. McKinsey).

## DISCLOSURES

No conflicts of interest, financial or otherwise, are declared by the authors.

## AUTHOR CONTRIBUTIONS

Y.L., H.K., J.W.E., X.C., and S.R.H. conceived and designed research; Y.L., J.P.J., D.M.E., R.M.B., and M.F. performed experiments; Y.L. and D.Y. analyzed data; Y.L. interpreted results of experiments; Y.L. prepared figures; Y.L. drafted manuscript; Y.L., D.Y., Y.Y., J.P.J., M.F., T.A.M., J.Y., J.W.E., X.C., and S.R.H., edited and revised manuscript; Y.L., H.K., D.Y., Y.Y., J.P.J., D.M.E., M.F., T.A.M., J.Y., J.W.E., X.C., and S.R.H. approved final version of manuscript.
